# Supplementary material for: Iron status and the risk of sepsis and severe COVID-19: a two-sample Mendelian randomization study
Source: Sci Rep. 2022 Sep 28;12:16157. doi: 10.1038/s41598-022-20679-6 (PMC9516524; doi:10.1038/s41598-022-20679-6)
Supplement: Supplementary file 1 — Supplementary Information. [file 41598_2022_20679_MOESM1_ESM.docx]

**Supplemental material**

Iron status and the risk of sepsis and severe COVID-19: A two-sample Mendelian randomization study

Randi Marie Mohus^1,2^ *

Helene Flatby^1^

Kristin V. Liyanarachi^1,3^

Andrew T. DeWan^4,1^

Erik Solligård^1^

Jan Kristian Damås^1,3,5^

Bjørn Olav Åsvold^6,7^

Lise T. Gustad^1,8,9^

Tormod Rogne^4,1,10^

**Supplemental Table S1: Included SNPs for all and sex-disaggregated analyses with explained variance and F-statistics for each iron biomarker**

|  |  | **All** | | | | **Women** | | | | **Men** | | | |
| --- | --- | --- | --- | --- | --- | --- | --- | --- | --- | --- | --- | --- | --- |
|  |  | Serum iron | TSAT | TIBC | Ferritin | Serum iron | TSAT | TIBC | Ferritin | Serum iron | TSAT | TIBC | Ferritin |
| No of SNPs | Sepsis | 14 | 10 | 15 | 33 | NA | NA | NA | NA | NA | NA | NA | NA |
|  | COVID-19 | 11 | 9 | 13 | 30 | 14 | 9 | 15 | 32 | 14 | 9 | 15 | 35 |
| Median variance explained (%) | | 1.8 % | 3.4 % | 2.2 % | 1.5 % | 2.0% | 3.5% | 1.9 % | 1.5% | 1.9% | 3.5% | 2.0% | 1.4% |
| Range Variance explained (%) | | 1.4– 7.5 % | 1.7– 9.7 % | 1.4–12.0% | 1.1– 3.7% | 1.1– 9.4% | 1.4– 10.5% | 1.3– 10.3% | 0.6– 4.9% | 0.9– 9.8% | 1.3– 12.5% | 0.5– 12.3% | 0.7– 3.8% |
| Median F-statistic | | 291 | 550 | 233 | 158 | 148 | 334 | 138 | 61 | 96 | 232 | 101 | 44 |
| Range F-statistic | | 226–1288 | 286– 1680 | 147– 1413 | 115– 397 | 79– 766 | 127–1076 | 90– 814 | 22–205 | 49– 543 | 82–924 | 27–702 | 22–122 |

TSAT; transferrin saturation, TIBC; total iron binding capacity, SNP; single nucleotide polymorphism, NA; not available

**Supplemental Table S2A: Included SNPs in MR analyses for sepsis and COVID-19 for each iron biomarkers**

| **Serum iron SNPs included in MR sepsis** | **Serum iron SNPs included in MR COVID-19** | **Serum iron SNPs included in MR COVID-19**  **WOMEN** | **Serum iron SNPs included in MR COVID-19**  **MEN** | **TSAT SNPs Included in MR sepsis** | **TSAT SNPs included in MR COVID-19** | **TSAT SNPs included in MR COVID-19**  **WOMEN** | **TSAT SNPs included in MR COVID-19**  **MEN** |
| --- | --- | --- | --- | --- | --- | --- | --- |
| rs1799945 | rs1799945 | rs1799945 | rs1799945 | rs1799945 | rs1799945 | 1) | 1) |
| rs1800562 | rs1800562 | rs1800562 | rs1800562 | rs1800562 | rs1800562 | rs1800562 | rs1800562 |
| rs57659670 | rs57659670 | rs57659670 | rs57659670 | rs57659670 | rs57659670 | rs57659670 | rs57659670 |
| rs855791 | NA | rs855791 | rs855791 | rs855791 | NA | rs855791 | rs855791 |
| rs2005682 | rs2005682 | rs2005682 | rs2005682 | rs2005682 | rs2005682 |  |  |
| rs4854760 | rs4854760 | rs4854760 | rs4854760 | rs4854760 | rs4854760 | rs4854760 | rs4854760 |
| rs9399136 | rs9399136 | rs9399136 | rs9399136 | rs9399136 | rs9399136 | rs9399136 | rs9399136 |
| rs13007705 | rs13007705 | rs13007705 | rs13007705 | rs13007705 | rs13007705 | rs13007705 | rs13007705 |
| rs7385804 | rs7385804 | rs7385804 | rs7385804 | rs7385804 | rs7385804 | rs7385804 | rs7385804 |
| rs12718598 | rs12718598 | rs12718598 | rs12718598 | rs3817672 | rs3817672 | rs3817672 | rs3817672 |
| rs7630745 | rs7630745 | rs7630745 | rs7630745 |  |  |  |  |
| rs77262773 | rs77262773 | rs77262773 | rs77262773 |  |  |  |  |
| rs35945185 | NA | rs35945185 | rs35945185 |  |  |  |  |
| rs2228145 | NA | rs2228145 | rs2228145 |  |  |  |  |

SNP; single nucleotide polymorphism, MR; Mendelian randomization, TSAT; transferrin saturation, NA; not available,

1) SNP removed from analysis for being palindromic.

**Supplemental Table S2B: Included SNPs in MR analyses for sepsis and COVID-19 for each iron biomarkers**

| **TIBC SNPs included in MR sepsis** | **TIBC SNPs included in MR COVID-19** | **TIBC SNPs included in MR COVID-19**  **WOMEN** | **TIBC SNPs included in MR COVID-19**  **MEN** | **Ferritin SNPs included in MR sepsis** | **Ferritin SNPs included in MR COVID-19** | **Ferritin SNPs included in MR COVID-19 WOMEN** | **Ferritin SNPs included in MR COVID-19 MEN** |
| --- | --- | --- | --- | --- | --- | --- | --- |
| rs1799945 | rs1799945 | 1) | 1) | rs1799945 | rs1799945 | rs1799945 | rs1799945 |
| rs1800562 | rs1800562 | rs1800562 | rs1800562 | rs1800562 | rs1800562 | rs1800562 | rs1800562 |
| rs57659670 | rs57659670 | rs57659670 | rs57659670 | rs57659670 | rs57659670 | rs57659670 | rs57659670 |
| rs855791 | NA | rs855791 | rs855791 | rs855791 | NA | rs855791 | rs855791 |
| rs3817672 | rs3817672 | rs3817672 | rs3817672 | rs1131262 | *rs1131262** | rs1131262 | 2) |
| rs4854760 | rs4854760 | rs4854760 | rs4854760 | rs10801913 | rs10801913 | rs10801913 | rs10801913 |
| rs9399136 | rs9399136 | rs9399136 | rs9399136 | rs1260326 | rs1260326 | rs1260326 | s1260326 |
| rs174546 | rs174546 | rs174546 | rs174546 | rs1250259 | rs1250259 | rs1250259 | rs1250259 |
| rs59950280 | rs59950280 | rs59950280 | rs59950280 | rs12693541 | rs12693541 | rs12693541 | rs12693541 |
| rs12693541 | rs12693541 | rs12693541 | rs12693541 | rs12807014 | rs12807014 | rs12807014 | rs12807014 |
| rs112727702 | *rs112727702** | rs112727702 | rs112727702 | rs12419620 | *rs12419620** | rs12419620 | rs12419620 |
| rs1132274 | *rs1132274** | rs1132274 | rs1132274 | rs2529440 | rs2529440 | rs2529440 | rs2529440 |
| rs17580 | rs17580 | 1) | 1) | rs36184164 | rs36184164 | rs36184164 | rs36184164 |
| rs1495743 | NA | 1) | 1) | rs17476364 | rs17476364 | rs17476364 | rs17476364 |
| rs6025 | rs6025 | rs6025 | rs6025 | rs6025 | rs6025 | rs6025 | rs6025 |
|  |  | rs46988 | rs46988 | rs601338 | rs601338 | rs601338 | rs601338 |
|  |  |  |  | rs34523089 | rs34523089 | rs34523089 | rs34523089 |
|  |  |  |  | rs3743171 | rs3743171 | rs3743171 | rs3743171 |
|  |  |  |  | rs13253974 | rs13253974 | rs13253974 | rs13253974 |
|  |  |  |  | rs4808802 | rs4808802 | rs4808802 | rs4808802 |
|  |  |  |  | rs4841429 | rs4841429 | rs4841429 | rs4841429 |
|  |  |  |  | rs4938939 | rs4938939 | rs4938939 | rs4938939 |
|  |  |  |  | rs55789050 | rs55789050 | rs55789050 | rs55789050 |
|  |  |  |  | rs6029148 | rs6029148 | rs6029148 | s6029148 |
|  |  |  |  | rs6757653 | rs6757653 | rs6757653 | rs6757653 |
|  |  |  |  | rs708686 | rs708686 | rs708686 | rs708686 |
|  |  |  |  | rs7865362 | rs7865362 | rs7865362 | rs7865362 |
|  |  |  |  | rs9921222 | rs9921222 | rs9921222 | rs9921222 |
|  |  |  |  | rs996347 | rs996347 | rs996347 | s996347 |
|  |  |  |  | rs3747602 | *rs3747602** | rs3747602 | rs3747602 |
|  |  |  |  | rs1542752 | *rs1542752** | rs1542752 | rs1542752 |
|  |  |  |  | rs34216132 | NA | 2) | rs34216132 |
|  |  |  |  | rs75965181 | NA | rs75965181 | rs75965181 |
|  |  |  |  |  |  |  | rs143041401 |
|  |  |  |  |  |  |  | rs3743171 |
|  |  |  |  |  |  |  | rs535064984 |
|  |  |  |  |  |  |  | rs551459670 |

SNP; single nucleotide polymorphism, MR; Mendelian randomization, TSAT; transferrin saturation, NA; not available,

1. SNP removed from analysis for being palindromic.
2. SNP not present in the sex specific iron biomarker GWAS.

**Supplemental Table S3: PhenoScanner results for included SNPs linked to other biological traits than iron status**

| **SNP** | **Trait** | **BETA** | ***P* value** |
| --- | --- | --- | --- |
| rs1250259 | Total cholesterol | 0.03 | 7.56e-06 |
| rs1250259 | LDL cholesterol | 0.03 | 1.46e-06 |
| rs1250259 | Blood pressure | -0.02 | 1.57e-11 |
| rs1260326 | Triglycerides | -0.12 | 4.00e-253 |
| rs1260326 | Total cholesterol | -0.05 | 3.00e-42 |
| rs1260326 | Diabetes 2 | 0.08 | 3.70e-09 |
| rs1260326 | Neutrophil count | -0.03 | 1.16e-19 |
| rs1260326 | Lymphocyte count | 0.03 | 2.00e-12 |
| rs1260326 | CRP | -0.07 | 5.00e-40 |
| rs12807014 | BMI | -0.02 | 1.49e-15 |
| rs34523089 | Monocyte count | -0.05 | 1.77e-21 |
| rs34523089 | Granulocyte count | 0.05 | 1.71e-26 |
| rs3743171 | Monocyte count | 0.04 | 6.31e-14 |
| rs3743171 | Granulocyte count | -0.04 | 7.13e-14 |
| rs3743171 | Neutrophile count | -0.02 | 1.96e-06 |
| rs3743171 | BMI | 0.01 | 6.50e-06 |
| rs4808802 | Total cholesterol | 0.03 | 3.27e-08 |
| rs4808802 | Granulocyte count | 0.02 | 4.72e-06 |
| rs55789050 | Diabetes 2 | -0.06 | 7.80e-07 |
| rs601338 | Total cholesterol | -0.03 | 2.41e-10 |
| rs174546 | Neutrophile count | 0.02 | 3.11e-10 |
| rs174546 | Monocyte count | -0.03 | 2.71e-14 |
| rs174546 | Granulocyte count | 0.03 | 5.03e-16 |
| rs174546 | Triglycerides | -3.82 | 5.00e-24 |
| rs174546 | Total cholesterol | 0.05 | 2.67e-37 |
| rs2228145 | IL-6 | NA | 2.00e-57 |
| rs2228145 | Coronary artery disease | 0.04 | 4.80e-14 |
| rs2228145 | CRP | 0.11 | 1.96e-10 |
| rs2228145 | Granulocyte count | 0.02 | 4.23e-07 |
| rs2228145 | Monocyte count | -0.02 | 8.94e-06 |
| rs35945185 | Lymphocyte count | -0.02 | 2.47e-06 |
| rs35945185 | Granulocyte count | -0.05 | 2.74e-36 |
| rs35945185 | Neutrophile count | -0.05 | 6.22e-36 |
| rs1799945 | Hypertension | -0.1 | 2.00e-10 |
| rs1799945 | HbA1c | 0.02 | 3.76e-19 |
| rs1800562 | HbA1c | -0.04 | 4.67e-28 |
| rs1800562 | Total cholesterol | -0.06 | 1.91e-12 |
| rs855791 | HbA1c | -0.02 | 3.44e-28 |
| rs17580 | Granulocyte count | -0.04 | 6.30e-06 |
| rs59950280 | Triglycerides | 0.04 | 1.00e-10 |
| rs59950280 | Coronary artery disease | 0.04 | 1.00e-06 |
| rs59950280 | Total cholesterol | 0.04 | 1.00e-10 |
| rs9399136 | White blood cell count | -0.05 | 1.65e-29 |

LDL; low density lipoprotein, CRP; C-reactive protein, BMI; body mass index, IL-6; Interleukin-6, HbA1c; glycosylated hemoglobin

**Supplemental Table S4: MR estimates for serum iron and ferritin on risk of sepsis after omitting SNPs related to white blood cell count and IL-6**

| **Iron biomarker** | **Number of SNPs** | **MR Methods** | **OR** | **95% CI** | ***P* value** |
| --- | --- | --- | --- | --- | --- |
| Serum iron | 10 | IWV | 1.16 | 1.03 – 1.32 | 0.019 |
| Ferritin | 30 | IWV | 1.05 | 0.88 – 1.24 | 0.61 |

SNP; single nucleotide polymorphism, OR; odds ratio, CI; confidence interval, IVW; inverse variance weighted

**Supplemental Table S5: MR estimates for serum iron on risk of being hospitalized with COVID-19 compared to non-hospitalized COVID-19 after omitting SNPs related to white blood cell count and IL-6**

| **Iron biomarker** | **Number of SNPs** | **MR Methods** | **OR** | **95% CI** | ***P* value** |
| --- | --- | --- | --- | --- | --- |
| Serum iron | 10 | IWV | 1.27 | 0.94 – 1.71 | 0.12 |
| Ferritin | 26 | IWV | 1.13 | 0.79 – 1.61 | 0.50 |

SNP; single nucleotide polymorphism, OR; odds ratio, CI; confidence interval, IVW; inverse variance weighted

**Supplemental Table S6: MR Egger intercept for all iron biomarkers on risk of sepsis**

|  | | **Risk of sepsis** | |
| --- | --- | --- | --- |
| **Iron biomarker** | **Number of SNPs** | **MR Egger intercept** | ***P* value** |
| Serum iron | 14 | -0.018 | 0.02 |
| TSAT | 10 | -0.013 | 0.15 |
| TIBC | 15 | -0.003 | 0.70 |
| Ferritin | 33 | -0.007 | 0.28 |

SNP; single nucleotide polymorphism, TSAT; transferrin saturation, TIBC; total iron binding capacity

**Supplemental Table S7: MR PRESSO results for all iron biomarkers on risk of sepsis**

|  | | **Risk of sepsis** | | |
| --- | --- | --- | --- | --- |
| **Iron biomarker** | **Number of SNPs** | **Global test** | **p-value** |  |
| Serum iron | 14 | 22.66 | 0.15 | No outliers detected |
| TSAT | 10 | 15.07 | 0.23 | No outliers detected |
| TIBC | 15 | 22.80 | 0.32 | No outliers detected |
| Ferritin | 33 | 40.72 | 0.23 | No outliers detected |

MR PRESSO; Mendelian randomization Pleiotropy RESidual Sum Outlier, SNP; single nucleotide polymorphism, TSAT; transferrin saturation, TIBC; total iron binding capacity

**Supplemental Table S8: MR Egger intercept for all iron biomarkers on risk of COVID-19 outcomes**

|  | | **Hospitalized COVID-19 vs non-hospitalized COVID-19** | | **Hospitalized COVID-19 vs population** | |
| --- | --- | --- | --- | --- | --- |
| Iron biomarker | Number of SNPs | MR Egger intercept | *P* value | MR Egger intercept | *P* value |
| Serum iron | 11 | -0.011 | 0.51 | 0.007 | 0.62 |
| TSAT | 9 | -0.0001 | 0.99 | 0.012 | 0.36 |
| TIBC | 13 | 0.008 | 0.60 | 0.004 | 0.71 |
| Ferritin | 31 | 0.004 | 0.79 | -0.003 | 0.78 |

SNP; single nucleotide polymorphism, TSAT; transferrin saturation, TIBC; total iron binding capacity

**Supplemental Table S9: MR PRESSO results for all iron biomarkers in risk of COVID-19 outcomes**

|  | | **Hospitalized COVID-19 vs non-hospitalized COVID-19** | | | **Hospitalized COVID-19 vs population** | | | | |
| --- | --- | --- | --- | --- | --- | --- | --- | --- | --- |
| Iron biomarker | Number of SNPs | Global test | p-value | Outlier test | Global test | p-value | Outlier test | Distortion test*  Beta p-value | |
| Serum iron | 11 | 9.30 | 0.74 | No outliers detected | 15.31 | 0.29 | No outliers detected |  |  |
| TSAT | 9 | 8.70 | 0.66 | No outliers detected | 12.73 | 0.34 | No outliers detected |  |  |
| TIBC | 13 | 18.30 | 0.32 | No outliers detected | 10.33 | 0.75 | No outliers detected |  |  |
| Ferritin | 31 | 20.01 | 0.93 | No outliers detected | 42.21 | 0.04 | 4 outliers detected | 356.87 | 0.12 |

MR PRESSO; Mendelian randomization Pleiotropy RESidual Sum Outlier, SNP; single nucleotide polymorphism, TSAT; transferrin saturation, TIBC; total iron binding capacity.

*) The distortion test was performed as both horizonal pleiotropy and outliers were detected for ferritin. The distortion test assesses the presence of differences in the causal estimate before and after outlier removal

**Supplemental Table S10: MR Egger intercept for all iron biomarkers in *women* for hospitalized COVID-19 vs non-hospitalized COVID-19**

| **WOMEN** | | **Hospitalized COVID-19 vs non-hospitalized COVID-19** | |
| --- | --- | --- | --- |
| **Iron biomarker** | **Number of SNPs** | **MR Egger intercept** | ***P* value** |
| Serum iron | 14 | -0.030 | 0.33 |
| TSAT | 9 | -0.003 | 0.91 |
| TIBC | 13 | 0.005 | 0.85 |
| Ferritin | 32 | -0.005 | 0.31 |

SNP; single nucleotide polymorphism, TSAT; transferrin saturation, TIBC; total iron binding capacity

**Supplemental Table S11: MR PRESSO results for all iron biomarkers in *women***

**Hospitalized COVID-19 vs non-hospitalized COVID-19**

| **WOMEN** | | **Hospitalized COVID-19 vs non-hospitalized COVID-19** | | |
| --- | --- | --- | --- | --- |
| **Iron biomarker** | **Number of SNPs** | **Global test** | ***P* value** |  |
| Serum iron | 14 | 23.09 | 0.11 | No outliers detected |
| TSAT | 9 | 8.31 | 0.53 | No outliers detected |
| TIBC | 13 | 28.69 | 0.26 | No outliers detected |
| Ferritin | 32 | 36.05 | 0.34 | No outliers detected |

MR PRESSO; Mendelian randomization Pleiotropy RESidual Sum Outlier, SNP; single nucleotide polymorphism, TSAT; transferrin saturation, TIBC; total iron binding capacity

**Supplemental Table S14: Bi-directional MR with MR estimates for the effect of sepsis on serum iron status**

|  | **Number of SNPs** | **MR Method** | **OR** | **95% CI** | ***P* value** |
| --- | --- | --- | --- | --- | --- |
| Sepsis on serum iron | 4 | IWV | 0.98 | 0.59 – 1.63 | 0.99 |

SNP; single nucleotide polymorphism, OR; odds ratio, CI; confidence interval, IVW; inverse variance weighted,

**Supplemental Table S15: Bi-directional MR with MR estimates for the effect of hospitalized COVID-19 vs non-hospitalized COVID-19 on serum iron status**

|  | **Number of SNPs** | **MR Method** | **OR** | **95% CI** | ***P* value** |
| --- | --- | --- | --- | --- | --- |
| COVID-19 on serum iron | 17 | IWV | 0.97 | 0.88–1.07 | 0.55 |

SNP; single nucleotide polymorphism, OR; odds ratio, CI; confidence interval. IVW; inverse variance weighted,

**Supplemental Figure S1: Workflow of the MR analyses**

**
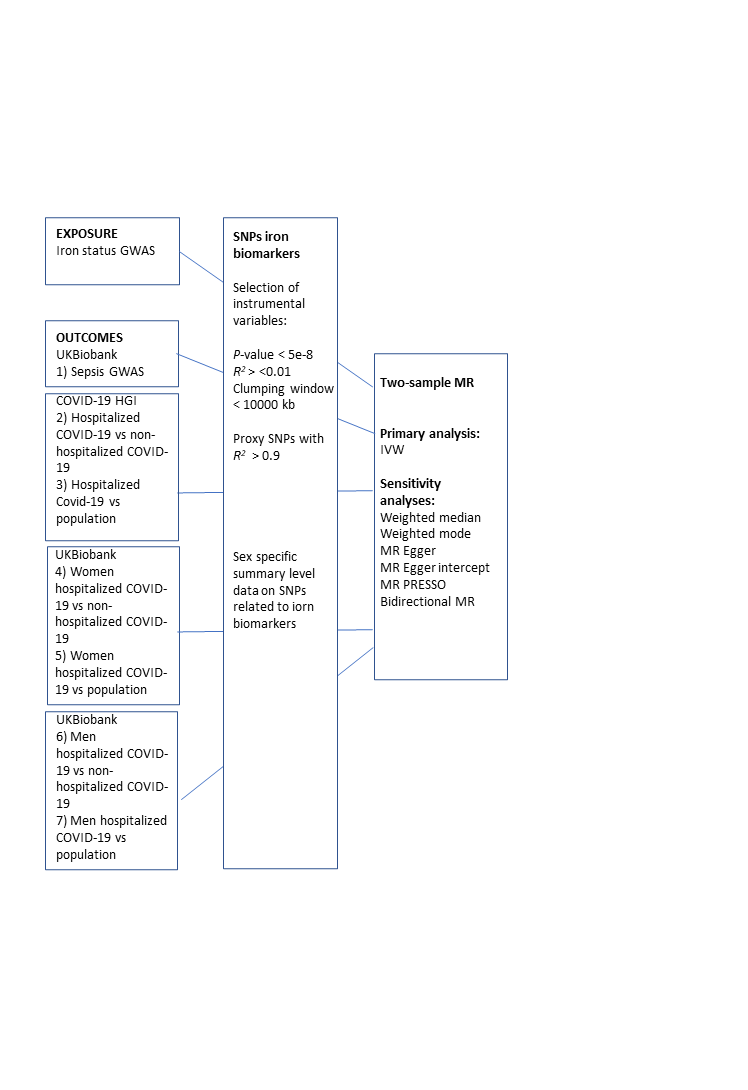
**

GWAS; genome wide association study, SNP; single nucleotide polymorphism, IVW; inverse variance weighted, MR PRESSO; Mendelian randomization Pleiotropy RESidual Sum Outlier

**Supplemental Figure S2 (A-D): Leave-one-out plots for the association between the iron biomarkers and sepsis**

A) Serum iron - sepsis B) TSAT – sepsis


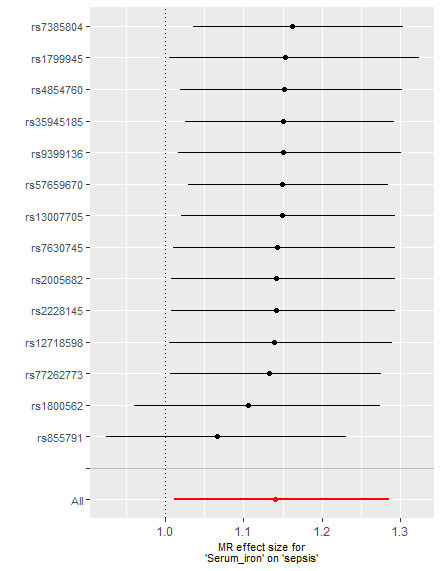

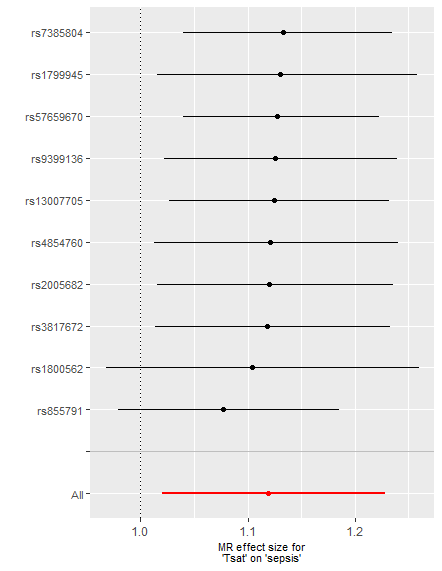


**Odds ratios with 95% CIs Odds ratios with 95% CIs**

C) TIBC – sepsis D) Ferritin – sepsis


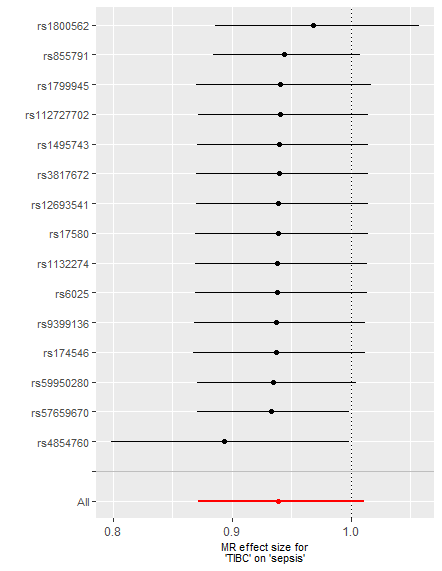

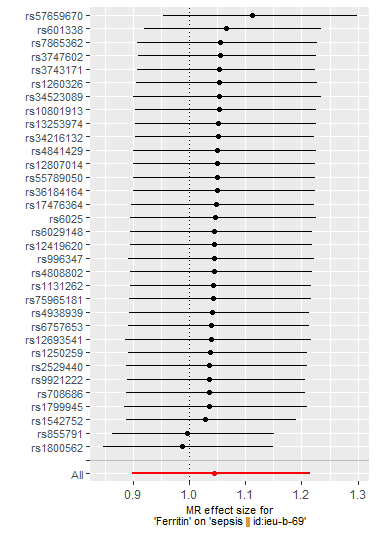


**Odds ratios with 95% CIs Odds ratios with 95% CIs**

**Supplemental Figure S3 (A-D): Leave-one-out plots for the association between the iron biomarkers and hospitalized COVID-19 vs non-hospitalized COVID-19**

A) Serum iron – hospitalized COVID-19 B) TSAT – hospitalized COVID-19


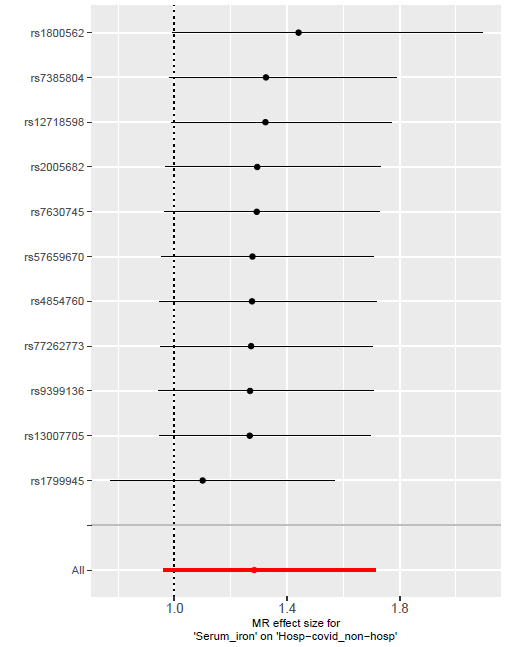

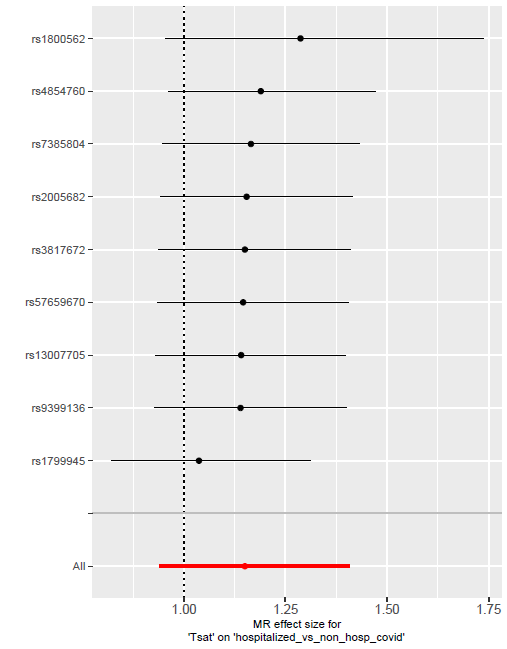


**Odds ratios with 95% CIs Odds ratios with 95% CIs**

C) TIBC – hospitalized COVID-19 D) Ferritin – hospitalized COVID-19


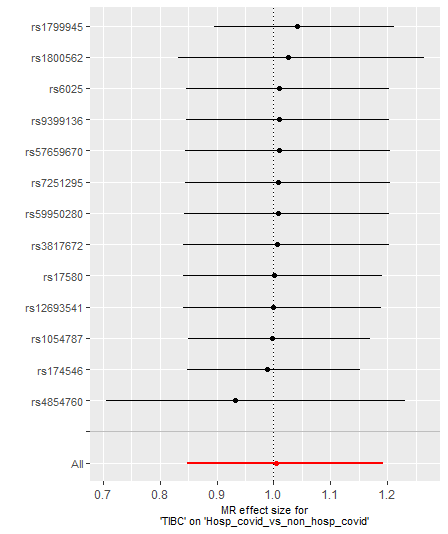

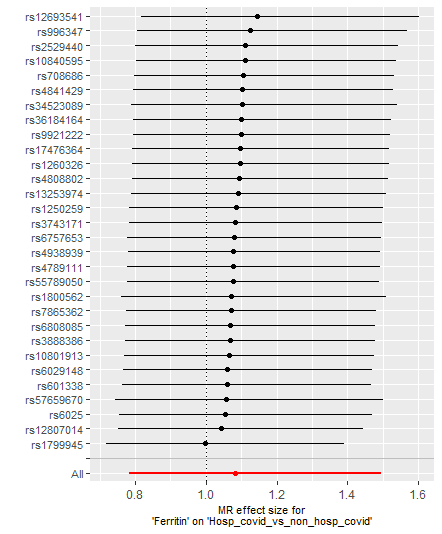


**Odds ratios with 95% CIs Odds ratios with 95% CIs**

**Supplemental Figure S4 (A-D): *Women* - Leave-one-out plots for the association between the iron biomarkers and hospitalized COVID-19 vs non-hospitalized COVID-19**

A) Serum iron B) TSAT


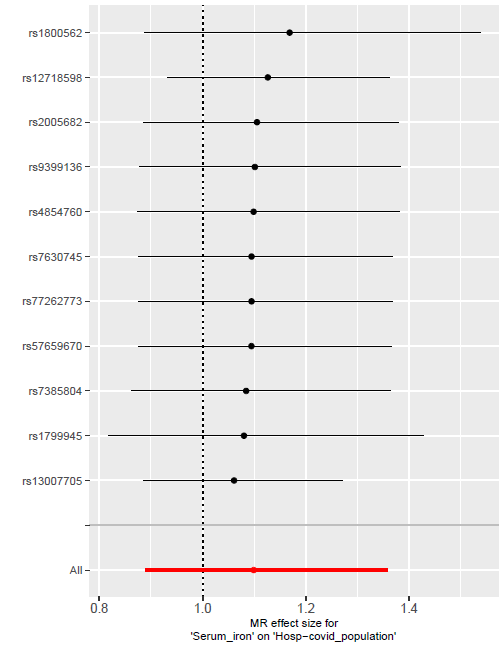

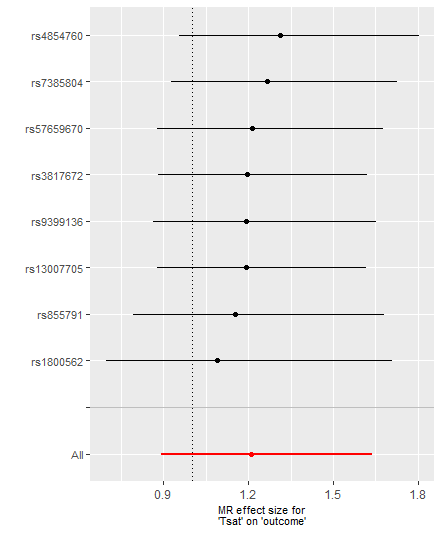


**Odds ratios with 95% CIs Odds ratios with 95% CIs**

C) TIBC D) Ferritin


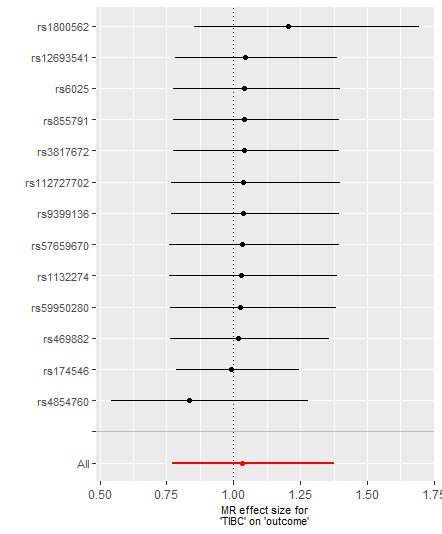

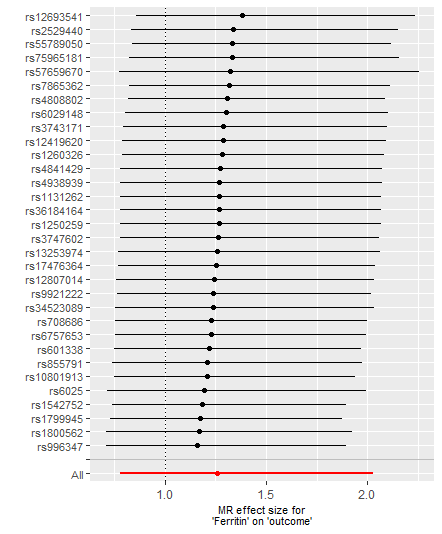


**Odds ratio with 95% CIs Odds ratio with 95% CIs** **Supplemental Figure S5 (A-D): *Men* - Leave-one-out plots for the association between the iron biomarkers and hospitalized COVID-19 vs non-hospitalized COVID-19**

A) Serum iron B) TSAT


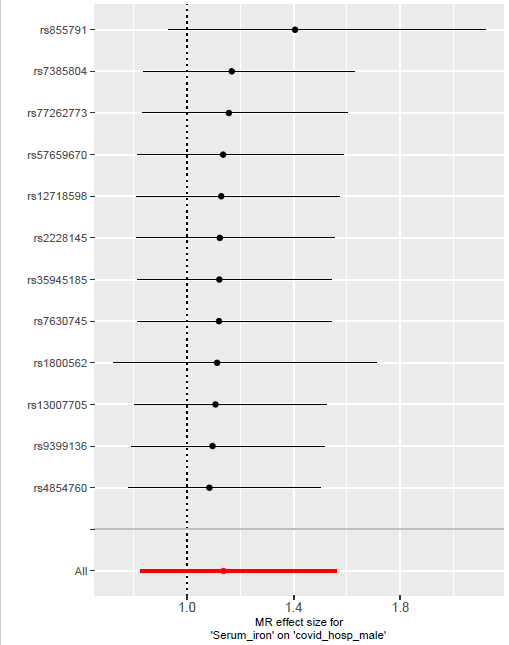

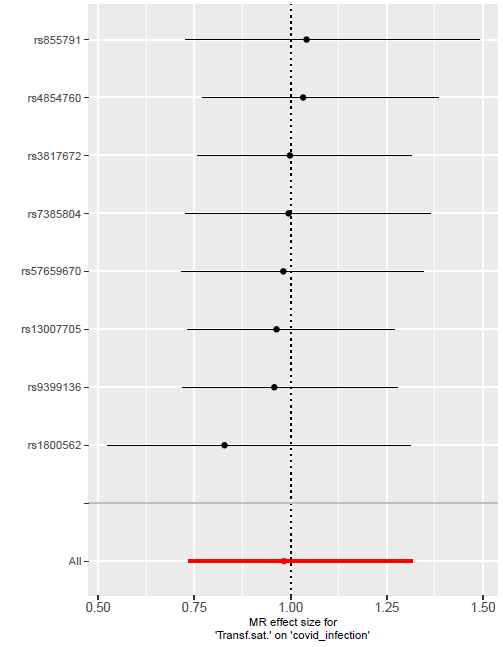


**Odds ratios with 95% CIs Odds ratios with 95% CIs**

C) TIBC D) Ferritin


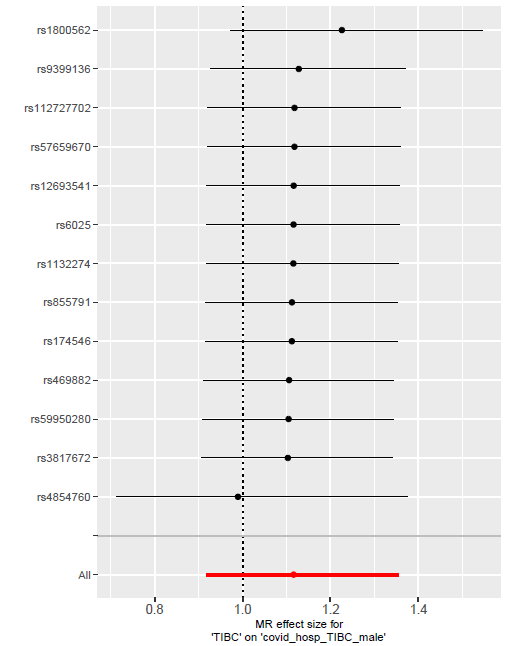

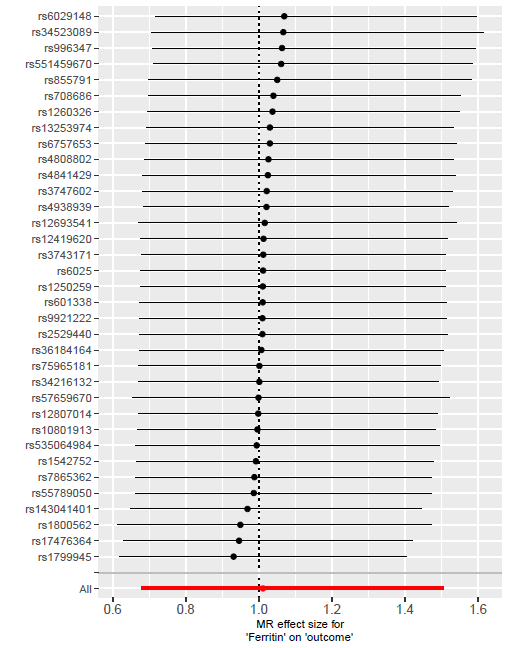


**Odds ratios with 95% CIs Odds ratios with 95% CIs**

**Supplemental Figure S6: Forest plot with MR estimates for risk of being hospitalized with COVID-19 compared with population**

**
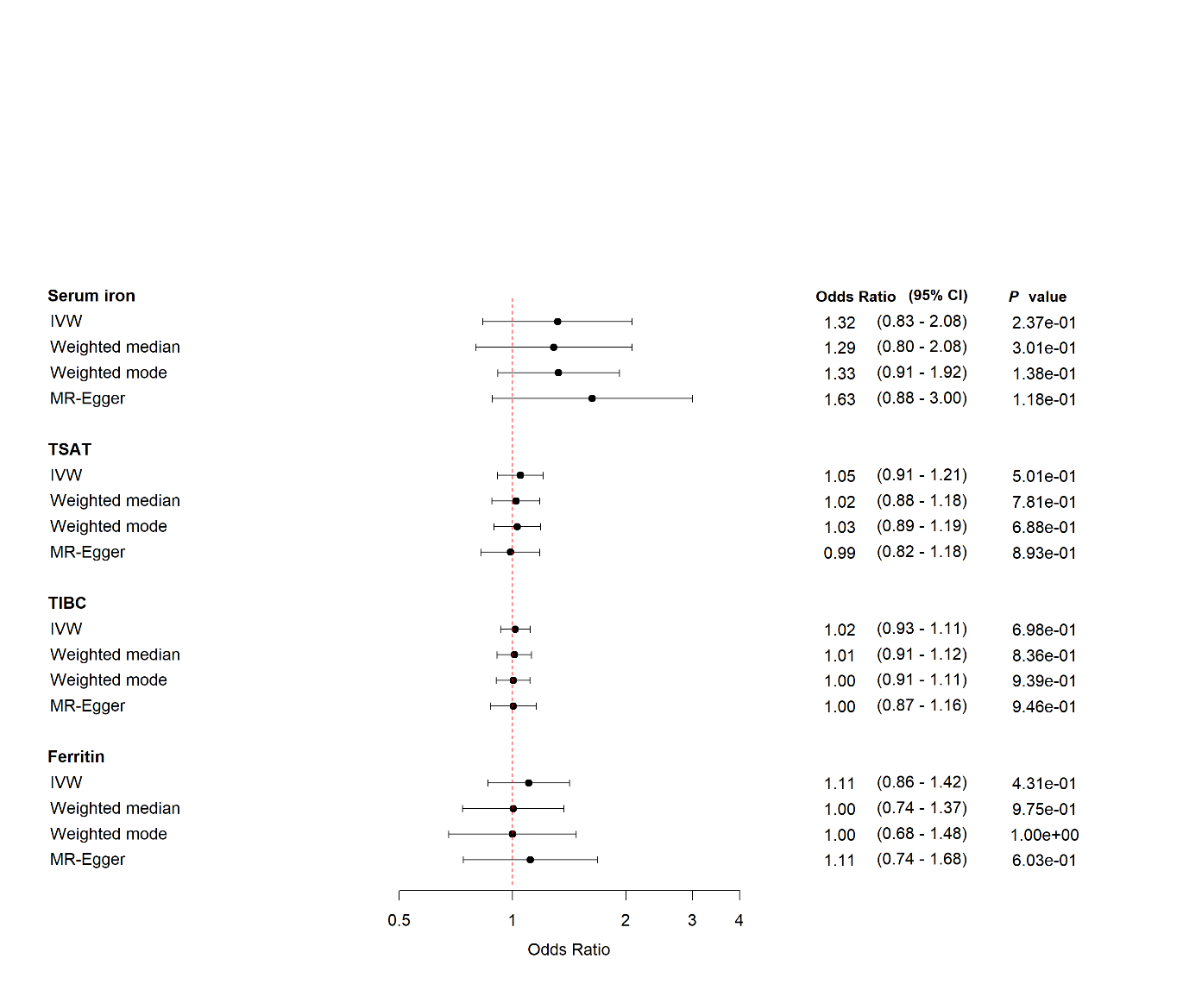
**

CI: Confidence Interval, TSAT; transferrin saturation, TIBC; total iron binding capacity, IVW: inverse variance weighted

**Supplemental Figure S7: Forest plot for *women* with MR estimates for risk of being hospitalized with COVID-19 compared with population**


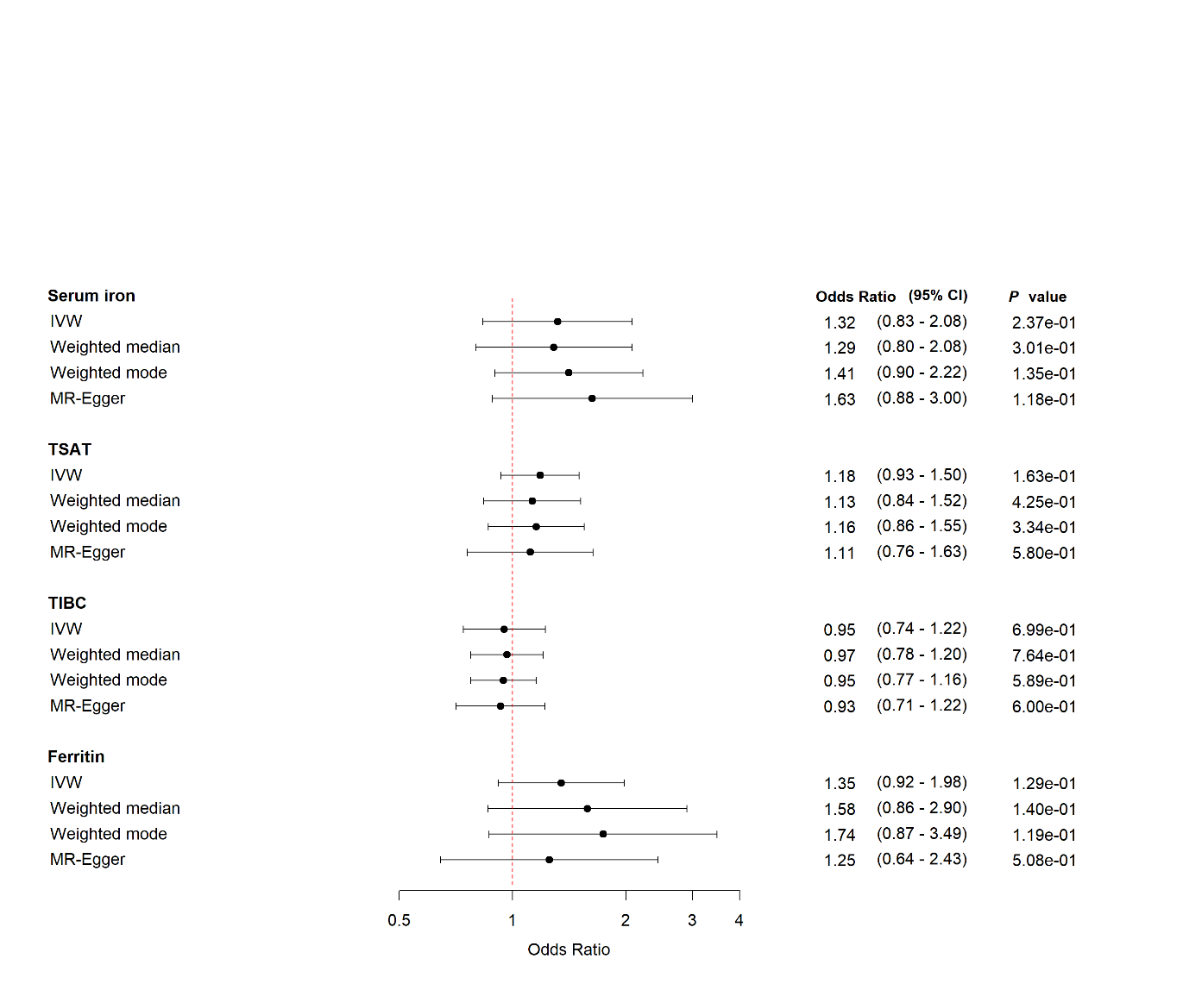


CI: Confidence Interval, Tsat; transferrin saturation, TIBC; total iron binding capacity

**Supplemental Figure S8: Forest plot for *men* with MR estimates for risk of being hospitalized with COVID-19 compared with population**


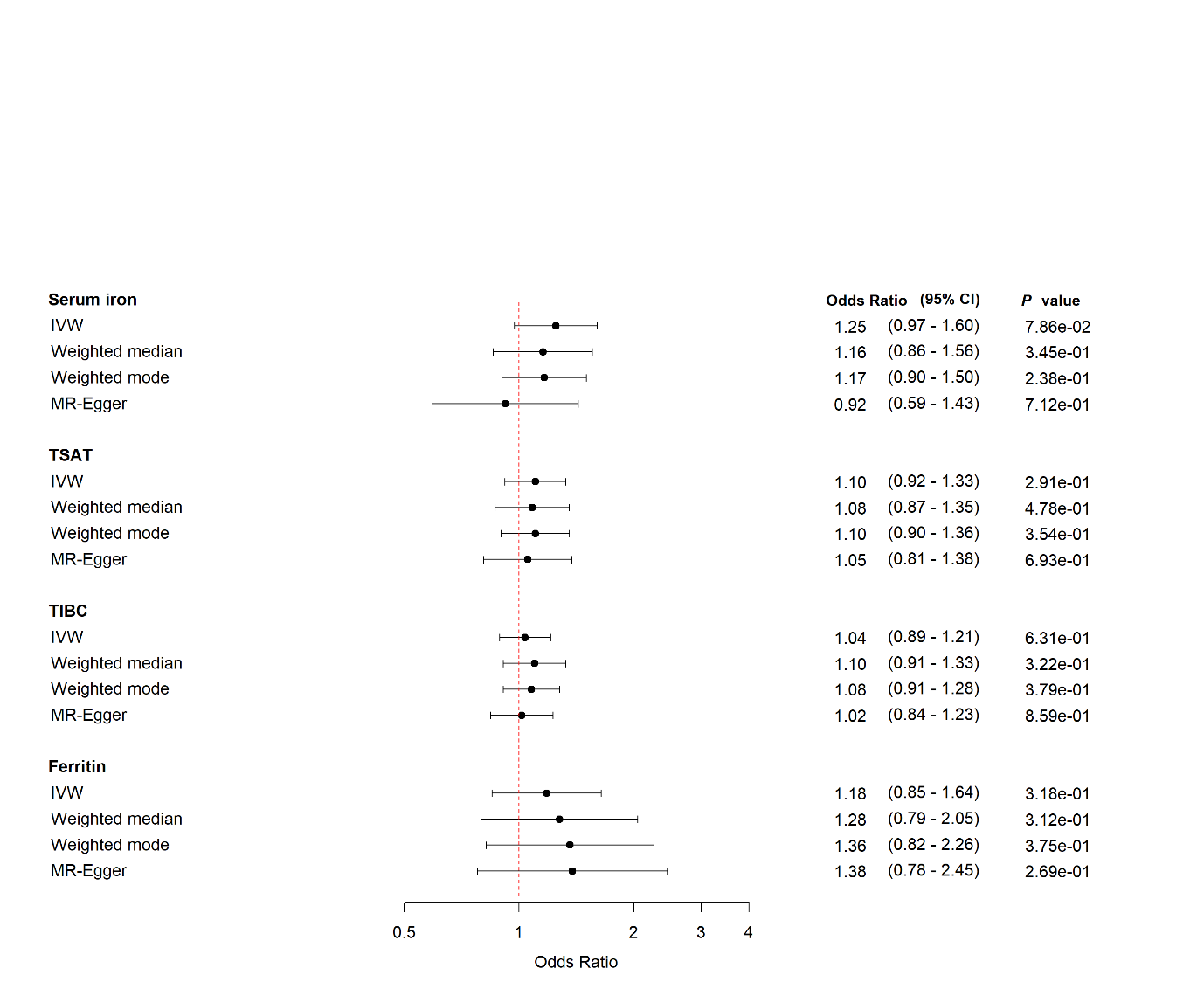


CI: Confidence Interval, Tsat; transferrin saturation, TIBC; total iron binding capacity
